# Alleviating anxiety and taming trauma: Novel pharmacotherapeutics for anxiety disorders and posttraumatic stress disorder

**DOI:** 10.1016/j.neuropharm.2023.109418

**Published:** 2023-01-06

**Authors:** Nicolas Singewald, Simone B. Sartori, Andreas Reif, Andrew Holmes

**Affiliations:** a Institute of Pharmacy, Department of Pharmacology and Toxicology, Center for Molecular Biosciences Innsbruck (CMBI), Leopold Franzens University Innsbruck, Innsbruck, Austria; b Department of Psychiatry, Psychosomatic Medicine and Psychotherapy, University Hospital, Goethe University Frankfurt, Frankfurt am Main, Germany; c Laboratory of Behavioral and Genomic Neuroscience, National Institute on Alcohol Abuse and Alcoholism, NIH, Bethesda, MD, USA

**Keywords:** Evidence-based drug discovery, Anxiolytic pharmacological therapy, Psychedelics, Cognitive enhancers of exposure therapy, RDoC

## Abstract

Psychiatric disorders associated with psychological trauma, stress and anxiety are a highly prevalent and increasing cause of morbidity worldwide. Current therapeutic approaches, including medication, are effective in alleviating symptoms of anxiety disorders and posttraumatic stress disorder (PTSD), at least in some individuals, but have unwanted side-effects and do not resolve underlying pathophysiology. After a period of stagnation, there is renewed enthusiasm from public, academic and commercial parties in designing and developing drug treatments for these disorders. Here, we aim to provide a snapshot of the current state of this field that is written for neuropharmacologists, but also practicing clinicians and the interested lay-reader. After introducing currently available drug treatments, we summarize recent/ongoing clinical assessment of novel medicines for anxiety and PTSD, grouped according to primary neurochemical targets and their potential to produce acute and/or enduring therapeutic effects. The evaluation of putative treatments targeting monoamine (including psychedelics), GABA, glutamate, cannabinoid, cholinergic and neuropeptide systems, amongst others, are discussed. We emphasize the importance of designing and clinically assessing new medications based on a firm understanding of the underlying neurobiology stemming from the rapid advances being made in neuroscience. This includes harnessing neuroplasticity to bring about lasting beneficial changes in the brain rather than – as many current medications do – produce a transient attenuation of symptoms, as exemplified by combining psychotropic/cognitive enhancing drugs with psychotherapeutic approaches. We conclude by noting some of the other emerging trends in this promising new phase of drug development.

## 1. Introduction

Psychiatric disorders are an enormous and growing burden on global health, with conditions related to stress and anxiety among the most common and costly causes of morbidity (Craske et al., 2017; Gustavsson et al., 2011; Penninx et al., 2021a). By some estimates, up to one third of people will present with an anxiety disorder at some point in their life (Kessler et al., 2012; Wittchen et al., 2011), with prevalence spiking of anxiety-related symptoms during the recent COVID-19 pandemic (Santomauro et al., 2021; Taquet et al., 2021).

The Diagnostic and Statistical Manual for Mental Health Disorders-5 (DSM-5) currently categorizes around a dozen types of anxiety disorder. Of these, the most common are generalized anxiety disorder (GAD), social anxiety disorder (SAD), panic disorder (PD), and various phobias including agoraphobia. This most recent iteration of DSM no longer groups post-traumatic stress disorder (PTSD) amongst the anxiety disorders and categorizes PTSD as Trauma and Stressor-Related Disorders. A similar distinction has been made in the current revision of the International Classification of Diseases (ICD-11; World Health Organization). Nevertheless, for the purposes of this review we have chosen to retain reference to ‘PTSD,’ as it remains familiar to most neuroscientists and neuropharmacologists outside the field and in light of the overlap between anxiety disorders and PTSD in terms of current pharmacotherapy and targets for future drug development.

Organizing anxiety disorders and PTSD into specific diagnostic categories defined by the presentation of symptoms brings clarity and consistency to clinical practice and can be a necessary step towards the approval and delivery of evidence-based therapeutic care. There has, however, been an increasing emphasis on capturing the dimensional and comorbid nature of psychiatric illness and the biological constructs associated with specific neurobehavioral domains (e.g., negative valence, social processes). This is exemplified by the National Institute of Mental Health’s (NIMH) Research Domain Criteria (RDoC) framework (Insel et al., 2010) and the Hierarchical Taxonomy Of Psychopathology (HiTOP) (Ruggero et al., 2019) which acknowledge that fear, anxiety and avoidance behavior are phenomena that cut across current diagnostic boundaries. This change in emphasis is aimed at improving the future identification of tractable, including pharmacological, treatments (Hariri and Holmes, 2015; van der Doef et al., 2018).

Despite these promising developments, there of course remains some major challenges for the field to address in order to realize the potential of targeted therapeutics (Scherf-Clavel et al., 2021). One ongoing debate revolves around the clinical relevance and validity of the commonly used experimental assays (primarily in male rodents) for anxiety and fear, given the subjective experience of these processes cannot be captured in rodents (Fanselow and Pennington, 2017; LeDoux and Pine, 2016; Nestler and Hyman, 2010). Relatedly, the fact that studies using these assays use male animals much more frequently than females -despite anxiety and PTSD being much more common and disabling in women than men (McLean et al., 2011) - has underscored the need to better consider sex as a biological variable in this and other areas of preclinical research (Arnegard et al., 2020; Shansky, 2020). We will not contribute to these important discussions here but suffice it to say recognizing these and other limitations is just first step towards finding solutions.

Our objective in the current review is to provide an update on the development of new pharmacological treatments for anxiety disorders and PTSD. As neuroscientists who are not, with one exception (A.R.), involved in clinical practice, we have endeavored to make the review accessible to a non-clinician audience looking for a snapshot of the main developments in the field. We are fully aware that psychotherapy, especially exposure therapy as a form of cognitive behavioral therapy, are highly effective in some forms of anxiety disorder and PTSD, depending on the severity of symptom and the type of psychological intervention. However, in this review we focus on pharmacological therapies (including pharmacological augmentation of psychotherapy) and refer the reader to other sources for information relating to psychological therapies (Alshahrani et al., 2022; Carpenter et al., 2018; Leichsenring et al., 2022). We begin by familiarizing the reader with the currently used drug treatments and note some of the approaches to expanding this treatment option-space. We then devote much of the remaining review to summarizing the status of clinical evaluation of potential medicines for anxiety and PTSD, which we group according to primary known neurochemical target. Lastly, we provide some concluding thoughts on the emerging trends and outlook for the field. For further discussion of this rapidly evolving literature, also on animal data as important tools for drug development in this field we refer the reader to complementary perspectives (Gasparyan et al., 2022; Sartori and Singewald, 2019).

## 2. Current pharmacological treatments and trends

Medication and cognitive behavioral therapy are the mainstays of treatment for the above conditions (Bandelow et al., 2022a, 2022b; Caldirola et al., 2021; Garakani et al., 2020; Gasparyan et al., 2022; Hoskins et al., 2021a; Locher et al., 2017; Slee et al., 2019; Williams et al., 2022). Among the most prescribed are selective serotonin reuptake inhibitors (SSRI) and selective serotonin-norepinephrine reuptake inhibitors (SNRI) (de Vries et al., 2018; Jakubovski et al., 2019). The therapeutic effects of these medications, as compared to placebo, has been demonstrated in randomized controlled trials (RCTs) (summarized in (Penninx et al., 2021a)), with effect sizes of around 0.4. However, while effective in many people, these drugs typically take weeks to produce beneficial effects and, in a sizeable proportion of individuals have side-effects such as nausea, gastro-intestinal symptoms, sexual dysfunction and changes in weight (Krystal et al., 2017). These unwanted side-effects notwithstanding, SSRIs (particularly sertraline) and (to a lesser extent) SNRIs are the current pharmacological treatments of choice in both primary and secondary care, and promoted as such in guidelines on the treatment of anxiety disorders from authorities such as the UK’s NICE (https://www.nice.org.uk/guidance/cg113) and Germany’s S3 (https://www.awmf.org/leitlinien/detail/ll/051-028.html). Added to the fact that SSRIs and SNRIs can be prescribed in a relatively simple dosing scheme, have limited potential for contraindications and are generally low cost, these drugs have become the standard of care in PD, SAD, GAD and PTSD.

Second line treatments, including monoamine oxidase inhibitors (MAO-I) and tricyclic and tetracyclic antidepressants (TCA) also target the monoamine system, but have somewhat different side-effects that limit their use; thus, these compounds are mainly reserved for very severe and/or treatment resistant cases in secondary or primary care. Pregabalin has, by contrast, a relatively benign side-effect profile, albeit with some potential for dependency, and is approved for the treatment of GAD. Though prescribed less often than SSRIs/SNRIs, pregabalin tends to be a more common option than two other licensed treatments for GAD, buspirone and opipramol. Off-label treatments used in anxiety disorders and PTSD include gabapentin and other anticonvulsants (e.g., topiramate), as well as second generation antipsychotics, such as quetiapine or risperidone (Garakani et al., 2022). These medications show efficacy in meta-analysis (de Moraes Costa et al., 2020), but second generation antipsychotics in particular can have adverse effects such as weight gain, metabolic syndrome and cardiovascular issues (Højlund et al., 2022). Nonetheless, low-doses of quetiapine are often used off-label as a mild anxiolytic, especially as an adjunct to other treatments.

There is little doubt that the benzodiazepines, such as diazepam and alprazolam, are a highly effective class of anxiolytics, but their long-term use is limited in particular by tolerance, dependence and abuse liability (Balon and Starcevic, 2020). These drugs can also produce cognitive impairments, such as amnesia, that can interfere with the processes that underlie cognitive-based therapies, such as the extinction and reconsolidation of fearful memories (Hart et al., 2014). Finally, though benzodiazepines produce rapid and often profound anxiolytic effects, these effects wane as the drug is eliminated from the body without the longer-term residual benefits that can be seen after a chronic course of, for example, an SSRI or SNRI. For these reasons, the unsupervised, long-term use of benzodiazepines is discouraged, though not without some controversy (Penninx et al., 2021b; Tibrewal et al., 2021).

Unfortunately, all of the current medications often only partially alleviate symptoms and provide only marginal benefit to a sizeable fraction of individuals with anxiety disorders and PTSD (Roy-Byrne, 2015), with varying rates in men and women. Sex differences in etiology and treatment response are increasingly discussed in the context of these disorders (Bangasser and Cuarenta, 2021; Bauer, 2023; Christiansen and Berke, 2020; Kundakovic and Rocks, 2022), but are still not sufficiently addressed. The limitations of efficacy of current treatments becomes more marked over time, with significant rates of long-term relapse (Bystritsky, 2006; Solis et al., 2021), particularly in those people with comorbid conditions such as major depressive disorder (MDD) (Ginsburg et al., 2018; Hofmeijer-Sevink et al., 2012). These limitations have clear and quantifiable consequences for morbidity and mortality, and the attendant economic and health care costs (Baxter et al., 2014; Higgins et al., 2021). However, while there is a compelling need for improved treatments for anxiety disorders and PTSD, the compound most recently developed for an anxiety disorder (i.e., GAD) since the benzodiazepines was the serotonin 5-HT1A receptor (R) agonist buspirone – and this was in the 1980s (reviewed in (Sartori and Singewald, 2019).

Most of the treatments for anxiety and PTSD that have emerged over the past few decades were initially approved for other indications, such as MDD (SSRIs, SNRIs), epilepsy (topiramate) and neuropathic pain (pregabalin). The paucity of new drugs certainly does not reflect a lack of interest or (intellectual and commercial) investment in identifying new medicines, both of which have been considerable (Griebel and Holmes, 2013; Pankevich et al., 2014). The reasons why these efforts have not translated into effective treatments are complex and not wholly clear. We have already mentioned moves towards giving more weight to neurobiological mechanisms over symptomatology, paving the way towards precision medicine approaches in psychiatry, and placing more emphasis on disease-dimensionality.

Relatedly, there are calls to rely less on explicitly atheoretical diagnostic systems, such as ICD-11 and DSM-5, which define disease as a summation of observable symptoms, and instead prioritize clinical evaluation of drugs with defined biological targets (e.g., NIMH FAST-FAIL and Target Engagement initiatives) (Grabb et al., 2020; Pizzagalli et al., 2020). It should be noted that these and related initiatives such as RDoC and HiTOP have not yet delivered tangible treatment outcomes. Some commentators have also suggested that these translational efforts can be aided with artificial intelligence (Gautam et al., 2022) and by designing more clinically relevant preclinical assays/models (Dunsmoor et al., 2022; Fraser et al., 2010; Richter-Levin et al., 2019; Singewald and Holmes, 2019; Tricklebank et al., 2021).

**Fig. 1.**
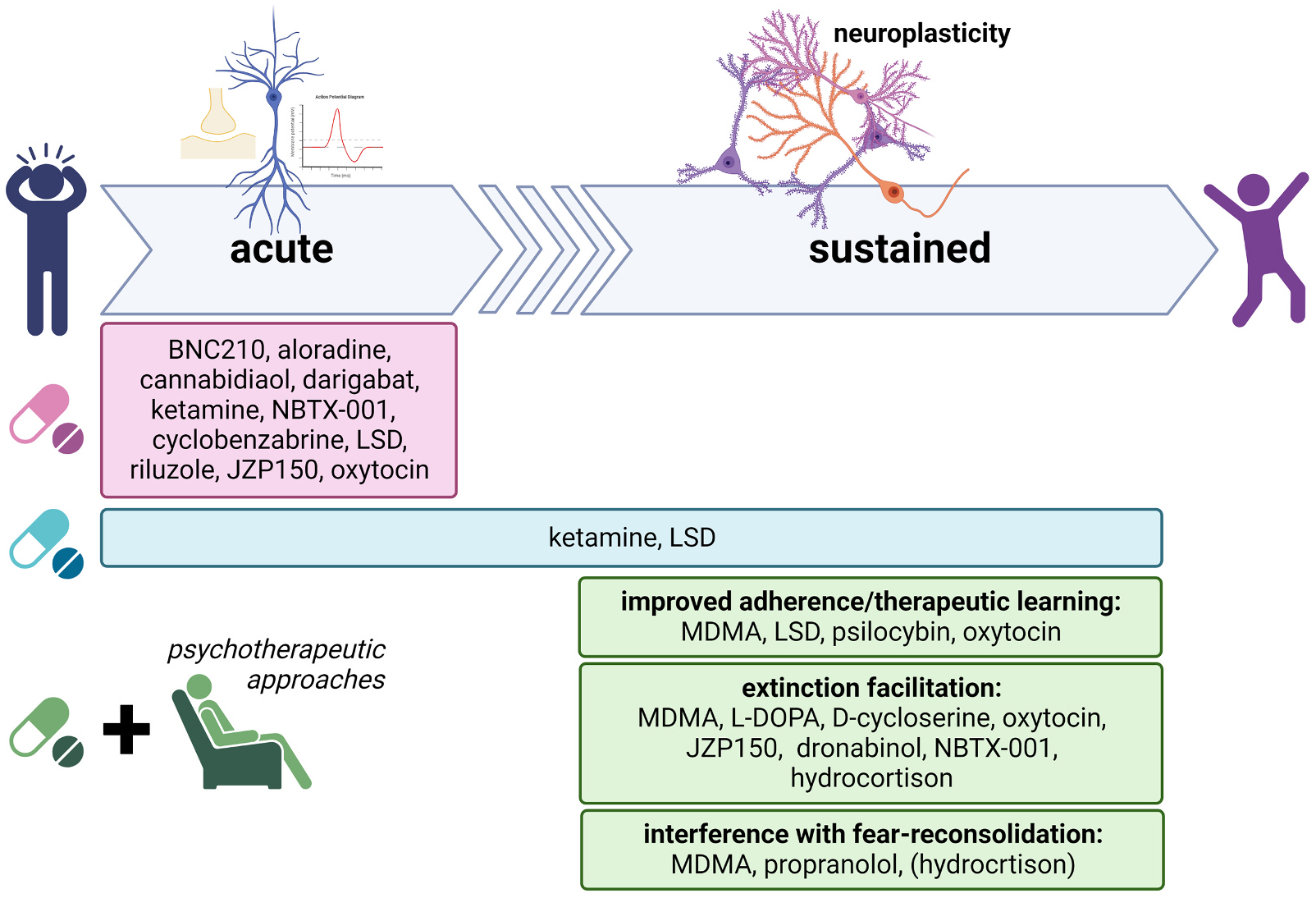
Examples of drugs in clinical development designed to produce acute and/or enduring therapeutic effects in anxiety disorders and/or PTSD.

We would agree with most of these developments, and especially the need to refocus both clinical evaluation and drug design on a foundation of neurobiology. The coming years will show whether these strategic shifts will bear fruit as improved treatments. For now, the field - largely based on the older model of assessing putative medications on their effects on symptoms – is arguably beginning to mature into some exciting developments that are currently being assessed at the level of clinical trials. We now turn to a summary of some of this large body of evidence, with further information on known mechanism(s) and clinical trial identifier provided in Tables 1–4. Fig. 1 shows examples of drugs in clinical development designed to produce acute and/or enduring therapeutic effects in anxiety disorders and/or PTSD.

## 3. Monoamines: Poly-pharmacology and psychedelics

The monoamine system has long been the major focus of efforts to develop effective pharmacotherapies for a broad range of affective disorders. Some of the most widely prescribed drugs in modern medicine, such as the SSRIs citalopram or sertraline, inhibit the reuptake of serotonin and, to varying degrees, norepinephrine (venlafaxine is currently the lead SNRI), among their various actions. However, the degree to which the monoamine-targeting actions of these drugs directly account for their therapeutic effects has still not been fully resolved (Murphy et al., 2021) (Holmes, 2008). This class of compounds has also come under criticism for being over-prescribed relative to their clinical efficacy, though this argument often understates the unequivocal benefits these drugs have given to many individuals (Cipriani et al., 2018).

Nonetheless, given the clinical and commercial success of monoamine-targeting drugs, such as the SSRIs there remains a lot of interest in identifying new anti-anxiety drugs that act by targeting various components of monoaminergic systems. There are several preliminary leads in this regard. For instance, several agents targeting specific components of the serotonergic system. These include vilazo-done (a 5-HT reuptake inhibitor and 5-HT1A-R partial agonist), vorti-oxetine (a 5-HT reuptake inhibitor, 5-HT3-R, 5-HT7-R and 5-HT1D-R antagonist, 5-HT1B-R partial agonist, and 5-HT1A-R agonist) – two drugs originally developed for MDD that have recently been investigated as potential treatments in anxiety disorders and PTSD. Some of these compounds have shown early signs of potential in clinical trials, though none have yet received FDA or EMA approval for these particular indications (for review see (Sartori and Singewald, 2019)). It seems unlikely, however, that these drugs outcompete older compounds, as their mechanism of action is not profoundly different to other antidepressants and their use in MDD has not suggested particularly marked anxiolytic effects.

Recent examples of drugs with multiple actions, some of which are monoaminergic, include AVN-101 and TNX-102 SL. AVN-101 is a structural analogue of dimebolin and has affinity for multiple 5-HT receptors and acts as an antagonist at dopamine and histamine receptors: it is currently in phase II (NCT04524975) and phase III (NCT04598867) evaluation for GAD. TNX-102 SL is a sublingual tablet formulation (to bypass first-pass hepatic metabolism) of cyclobenzaprine hydrochloride that is being investigated for its potential utility in PTSD (NCT05372887). TNX-102 SL is an example of a drug with a broad mechanistic profile that includes antagonist activity at the 5-HT2A-R, α1-adrenergic, histaminergic H1, and muscarinic M1 receptors. There is also ongoing phase I study (NCT05363839) investigating ACH 000029 in healthy participants (with a view to use in GAD and PTSD), a compound that acts as an agonist at 5-HT1A-R, a partial agonist at 5-HT1D-R and an antagonist at 5-HT2A-R and α-adrenergic (α−1A, 1B and 1D) receptors. The antipsychotic 5-HT2A-R inverse agonist/antagonist, pimavanserin, is being clinically assessed for its ability to alleviate insomnia and/or nightmares in PTSD – symptoms that individuals report as being highly disruptive to well-being. The α2-adrenergic receptor agonists dexmedetomidine and clonidine, which decrease norepinephrine release and sympathetic tone, are also being tested for their effects on these symptoms.

A handful of drugs which are used as medications in psychotic disorders have attracted some interest for their possible utility in anxiety disorders and PTSD. Among these is brexpiprazole, a partial agonist at D2-R and 5-HT1A-R and an antagonist at 5-HT2A-R and other serotonin receptors. Brexpiprazole is being evaluated alone and in combination with sertraline in a large multi-center, randomized PTSD trial (NCT04174170, NCT03033069). Quetiapine, which has demonstrated anxiolytic properties, blocks 5-HT2A-R and D2-R and has partial agonist effects at 5-HT1A-R, is currently being investigated as a treatment to facilitate the efficacy of exposure therapy in PTSD, with the idea that it may reduce anxiety and irritability to promote therapeutic engagement (NCT04280965).

The strategy of enhancing psychotherapy with psychotropic medication has become increasingly popular in the recent years, especially for the treatment of PTSD. Table 4 provides an overview of examples of drug-assisted psychotherapy. In principle, this strategy could represent a paradigm shift in the treatment of mental disorders and help reconcile pharmacological and psychological treatment approaches. However, there is still a need for well-controlled combination therapy studies that vary the timing and sequencing of both treatment modalities. Such studies can serve to identify the optimal parameters for combing drug treatment with psychological intervention, and ideally should be informed by known neurobiological and psychological mechanisms. An example is L-DOPA (Levodopa), the canonical dopamine precursor medicine for Parkinson’s disease, which has been examined for its possible use as adjunct to exposure therapy in PTSD. Another example is work investigating how best to time administration of beta-blockers, such as propranolol, relative to fear memory reactivation in order to disrupt (traumatic) memory reconsolidation (Raut et al., 2022a). This idea is based on the finding that memory transiently enters a labile state following retrieval, allowing unwanted memories to be revised as they are reconsolidated (Brunet et al., 2018). However, the usefulness of propranolol in this respect is still a matter of debate (Steenen et al., 2022).

The goal of exposure-based therapy is to reduce anxiety to feared situations, emotions, and memories by confronting the individual (directly or through visualisation) with these triggers and enabling reprocessing of their association with threat in a safety context (Foa and McLean, 2016; Huang et al., 2022; McLean et al., 2022; van Loenen et al., 2022). Data from rodent and human assays for fear extinction have shown that L-DOPA promotes long-term extinction, including in models of impaired extinction (Esser et al., 2021; Gerlicher et al., 2019; Haaker et al., 2013; Whittle et al., 2016). Based on these intriguing cross-species findings, coupled with evidence of augmented amygdala encoding of extinction in women with PTSD (Cisler et al., 2020), L-DOPA is currently being studied as an exposure therapy adjunct in a 120-participant phase II PTSD trial (NCT04558112) that examines symptom alleviation and, as measured by functional brain imaging, resting state brain activity. The hypothesis is that L-DOPA will boost the consolidation of extinction memory formed during exposure and produce associated changes in dopamine-related neural network activity. Again, proper timing and synchronization of drug and psychological treatment will be crucial to demonstrate efficacy.

### 3.1. Serotonergic psychedelics and entactogens

The future of targeting the monoamine system to alleviate anxiety may not only entail designing novel multi-pharmacology agents and repurposing existing drugs for other indications. A notable recent trend has been the therapeutic potential of psychedelic drugs, which have prominent serotonergic effects. Psychedelic agents comprise different classes defined by their chemical structure and pharmacological profiles (Inserra et al., 2021; McClure-Begley and Roth, 2022; Vollenweider and Smallridge, 2022). The dissociative hallucinogen, ketamine, which is sometimes considered a psychedelic, will be discussed below in the context of the glutamate system where its main mechanism of action rests.

Broadly speaking, there are the ‘classic’ serotonergic hallucinogens, that act as 5-HT2A-R agonists (among other 5-HT receptor actions) and include 4-phosphoryloxy-N,N-dimethyltryptamine (psilocybin), lysergic acid diethylamide (LSD) and N,N-Dimethyltryptamine (DMT, aka ayahuasca) and 5-methoxy-N,N-dimethyltryptamine (5-MeO-DMT). The entactogens, such as 3,4-methylenedioxy-methamphetamine (MDMA), which are mixed serotonin/dopamine/norepinephrine reuptake inhibitors/releasers with additional effects on oxytocin and prolactin, belong to the group of recreational illicit drugs which are tested for a potential therapeutic use in combination with psychotherapy. Their effects in the human brain are just beginning to be revealed (Drew, 2022). The clinical data on the utility of these drugs however largely pertains to MDD and remains very limited for anxiety and PTSD (see below and for review (McClure-Begley and Roth, 2022; Nutt and Carhart-Harris, 2020)). Research on the therapeutic potential of 5-MeO-DMT is at an early stage with preliminary evidence showing this drug can increase mindfulness and reduce anxiety in healthy participants (reviewed in (Reckweg et al., 2022)).

Results of trials with psilocybin or LSD (including the associated preparations COMP360 and MM-120, respectively) have been published for PTSD and GAD, but thus far been primarily related to ‘end-of-life anxiety’ in individuals with severe physical illnesses and a palliative prognosis (Fuentes et al., 2020; Grob et al., 2011; Kelmendi et al., 2016; Schimmel et al., 2022; Weston et al., 2020; Yu et al., 2021). There is a phase I/II trial on the effects of combining a single-dose of psilocybin with psychotherapy in frontline healthcare workers suffering burnout (NCT05163496). Relatedly, preclinical work suggests that a combination of psychedelic administration with extinction can have additive effects on this form of learning (Catlow et al., 2013).

At the neural level, studies in healthy participants have shown that psilocybin and LSD alters various measures of functional brain connectivity, including increased default mode connectivity and reduced amygdala reactivity, in a manner associated with reduced distress and increased mindfulness (Carhart-Harris et al., 2013; Daws et al., 2022; Preller et al., 2018, 2020, reviewed in (Averill and Abdallah, 2022). Of further note, these effects are related to changes in cortical 5-HT1A-R and 5-HT2A-R gene expression and can be blocked by the 5-HT2A-R antagonist ketanserin (Preller et al., 2017, 2018, 2020). Given these data identify 5-HT2A-R as at least one key mechanism of action of these drugs, they point the way to targeting this receptor subtype as a future path to investigating whether non-hallucinogenic analogs of psychedelics with a similar mode of action can retain therapeutic value.

MDMA has been granted Breakthrough Therapy Designation by the FDA (‘a process designed to expedite the development and review of drugs’) for drug-assisted psychotherapy in PTSD. When given as an adjunct to multiple psychotherapy sessions, MDMA has been shown in meta-analyses to reduce PTSD symptoms (Hoskins et al., 2021b; Mithoefer et al., 2019; Smith et al., 2022). Most notably, a randomized, double-blind and placebo-controlled phase III study (MAPP1) concluded that MDMA was highly efficacious in individuals with severe PTSD (Mitchell et al., 2021). Promisingly, these effects appear to generalize to treatment-resistant PTSD (Illingworth et al., 2021). A recent confirmatory phase III study (MAPP2) involving participants with moderate PTSD was recently concluded (NCT04077437), but results had not been reported at the time of writing. As with any nascent clinical literature, these exciting findings should be tempered by bearing in mind that additional studies of safety (including abuse liability) are still awaited (Krystal et al., 2021).

The mechanisms accounting for the clinical effects of MDMA remain obscure. The compound is the psychoactive component of drugs recreationally taken as ‘ecstasy’ or ‘molly,’ and reported by these users to reduce anxiety, increase sociability, empathy and trust, and produce other states that might facilitate receptiveness to psychotherapy, such as increased introspection and self-insight (Nichols, 2022). There is a view that by producing these altered states, MDMA and hallucinogenic drugs may be well-suited as adjuncts to psychotherapeutic approaches, including exposure-based therapies (Feduccia and Mithoefer, 2018; Glavonic et al., 2022). Two open-label phase II trials are planned to assess MDMA-assisted group therapy for PTSD (NCT05173831) and, separately, for SAD (NCT05138068) - which aligns with MDMA’s aforementioned pro-social effect. Of further relevance in this context are preclinical data in rodents and human showing that MDMA facilitates fear extinction (Maples-Keller et al., 2022a; Vizeli et al., 2022; Young et al., 2017). While this pro-extinction effect is one that is shared with SSRIs in rodent studies (Gunduz-Cinar et al., 2016; Karpova et al., 2011), the utility of a combined SSRI-extinction approach has not been borne out by clinical data (Popiel et al., 2015; Rauch et al., 2019) and it remains to be seen whether this also turns out to be the case with MDMA.

In addition to increasing monoamine levels, MDMA affects hormone levels (oxytocin, cortisol) and interacts with other downstream signaling molecules (e.g., BDNF) that can modulate emotional memory circuits (Glavonic et al., 2022). In fact, a feature common to the psychedelics is that they alter functional interconnectivity between certain brain regions and to affect changes related to neuroplasticity (e.g., (Daws et al., 2022; de Vos et al., 2021; Ly et al., 2018). This has led some authors to refer to the class as ‘psychoplastogens’ (Vargas et al., 2021), and others to posit that these drugs produce beneficial effects in PTSD by modifying trauma-related memory (Raut et al., 2022b). More generally, the notion that therapeutically co-opting neuroplasticity for therapeutic gain, by targeting defined neurochemical, genetic and epigenetic mechanisms, is gaining traction (Kraus et al., 2017; Luchkina and Bolshakov, 2018; Mishra et al., 2021).

A general issue related to MDMA and the psychedelics, is these drugs have been primarily tested in combination with psychotherapy, based on the notion that they enhance acceptance of and compliance with psychotherapy to thereby enhance the long-term therapeutic efficacy (Reiff et al., 2020). As noted above, this sort of combined approach makes it difficult to assess the effects of the combination and the effects of the drug *per se*, separate from the known benefits of psychotherapy. Appropriately designed, multi-arm clinical trials will be needed to dissociate the effects of joint therapy such as this. Given setting in which psychedelics are administered and the individual’s mindset at the time of treatment are thought to be important for therapeutic outcome (Hartogsohn, 2016), the influence of these factors also requires clarification. Notwithstanding, if the clinical effectiveness of this approach however matches the current optimism from some authorities in the field, psychedelic-assisted therapy (aka ‘psychedelic medicine’) could represent a major shift in how the treatment of anxiety disorders is conceptualized and delivered (Felsch and Kuypers, 2022; King and Hammond, 2021; Knudsen, 2022; McClure-Begley and Roth, 2022). While the approach holds much promise and could potentially be transformative, the jury remains out in lieu of the findings of rigorous clinical evaluation. If the results do prove to be positive and the drugs are licensed only for use when combined with psychotherapy, it could be challenging to ensure their use is restricted to well-controlled therapeutic settings.

## 4. GABA: mitigating side-effects through subunit-selectivity

The γ-aminobutyric acid (GABA) system is the prototypical anxiolytic target and recent GWAS studies further support the importance of GABA signaling in anxiety disorders at many levels (PGC-ANX GWAS meta-analysis, unpublished (Hettema et al., 2022)). Drugs such as diazepam have been synonymous with reducing anxiety since the benzodiazepines were found to have rapid anti-anxiety properties in the 1950s (Sternbach, 1979). Benzodiazepines act as positive allosteric modulators (PAM) of the GABA_A_-R - a pentameric ligand-gated ion channel composed of varying combinations of subunits (α,β,γ,δ,ρ,ε,θ, and π). However, these modulatory actions dose-dependently produce sedation, motor impairment, hypnosis and anesthesia, as well as dependence in some people and withdrawal problems following prolonged use. Hence, the utility of benzodiazepines as long-term therapeutics for anxiety and PTSD is quite limited and, as mentioned above, comes with the potential for abuse. These constraints led to a significant push to design novel GABAkines – compounds which act at specific GABA_A_-R subunits and thereby promoting anxiolytic effects while minimizing undesirable behavioral effects (Cerne et al., 2022; Chen et al., 2019).

Unfortunately, despite promising preclinical studies elegantly designed to decipher the specific subunits responsible for anxiety (α2 and α3 may be particularly key) versus other discrete behaviors (Castellano et al., 2021; Dias et al., 2005; Engin et al., 2018), no GABAkines have yet made it to later stages of clinical development for anxiety or PTSD (Cerne et al., 2022; Witkin et al., 2022). Notwithstanding these disappointments, there continues to be efforts in this area. A phase II trial for the GABA_A_-R PAM, PRAX-114, is currently underway for PTSD (NCT05260541) (although negative trial results were recently reported for the drug in MDD), and the α2/3/5-selective GABA_A_ PAM, CVL 865 (darigabat), is being studied following a positive phase I clinical trial showing attenuation of CO_2_ inhalation-induced panic.

Neuroactive steroids (neurosteroids), acting as endogenous GABA_A_-R PAMs, represent another potential route to achieving anxiolytic effects through the GABA system (Castellano et al., 2021; Longone et al., 2011; Zorumski et al., 2019). Brexanolone is an intravenous formulation of the neurosteroid, allopregnanolone, that is FDA approved for postpartum depression and now being evaluated for PTSD in a small open-label phase IV trial (NCT05254405) although the route of administration is impractical. An alternative option is an oral neurosteroid, zuronalone, which is currently being evaluated for MDD and postpartum depression. Another neurosteroid, aloradine (PH94B, fasedienol) is posited to act as a vomeropherine at chemosensory receptors in the vomeronasal organ and, via the activation of olfactory bulb neurons connecting to emotion-regulating areas of the brain. Aloradine has shown rapid-onset beneficial effects against SAD symptoms in a randomized, multi-center study when delivered in aerosol form as a nasal spray (Liebowitz et al., 2014, 2016). These results are being followed-up in phase III trials for SAD (NCT04754802, NCT05030350) and a phase II trial for adjustment disorder with anxious mood (NCT04404192). Finally, although there has been a view that activation of GABA signaling (e.g., via benzodiazepines) can – possibly through amnestic effects – oppose the benefits of extinction/exposure (Otto et al., 2010), a single intravenous dose of allopregnanolone is being assessed for its ability to promote extinction/reconsolidation in a double-blind, randomized phase II trial for PTSD (NCT04468360) (for further discussion of this area (Rasmusson et al., 2022)).

## 5. Glutamate: Counting on ketamine?

Glutamate – the major excitatory CNS neurotransmitter – exerts its action through three types of ionotropic receptors (*N*-methyl-D-aspartate (NMDA-R), alpha-amino-3-hydroxy-5-methyl-r-isooxazolepropionic acid (AMPA-R) and kainate) and eight metabotropic glutamate receptor (mGluR) subtypes. Stress triggers glutamate release, initiating a cascade of changes in synaptic plasticity, gene expression and DNA methylation and brain cell (neuronal and glial) remodeling that are accompanied by alterations in cognitive function and emotional behaviors (Popoli et al., 2022; Sanacora et al., 2022). A long-standing view is that excessive glutamate activity and associated excitatory-inhibitory imbalance contributes to the pathophysiology of anxiety and PTSD and, consequently, that glutamate-targeting drugs could be of significant therapeutic value (Ferraguti, 2018; Nasir et al., 2020; Popoli et al., 2022).

### 5.1. Ketamine

The enormous therapeutic interest in ketamine, a racemic drug consisting of the two pharmacologically active compounds arketamine and esketamine, is well-known. To-date, however, the clinical evidence still primarily relates to MDD and bipolar depression (Krystal et al., 2019, 2020): the ketamine-derived drug, esketamine, delivered as a nasal spray, is now FDA-approved for MDD, and also approved by the EMA for treatment-resistant depression. There is currently strong interest in the possibility of ketamine as a new medication for PTSD (Feder et al., 2020; Sartori and Singewald, 2019; Silote et al., 2020). Some models attribute the rapid-acting antidepressant properties of ketamine to the blockade of NMDA-Rs localized on GABA interneurons in the medial prefrontal cortex (PFC), resulting in increased glutamate and associated neuroplastic changes mediated by neurotrophic factors such as BDNF (Gerhard et al., 2020; Hess et al., 2022; Lavender et al., 2020).

Current mechanistic schemes do not discount the potential contribution of ketamine and its metabolites effects on non-glutamatergic systems (e.g., monoamine and opioid) – highlighting the fact that the drug’s mechanism of action is not yet fully understood. The off-label use of ketamine (i.v. or s.c.) has increased rapidly since the last 15 years, especially in the US where ‘ketamine clinics’ have been established that provide ketamine infusions, but little other care. The introduction of ketamine (especially for treatment-resistant depression) has been at a slower pace in most other countries, despite acknowledgement of its value for the treatment of MDD/treatment-resistant depression in secondary and especially tertiary care - as underscored by the clinical introduction of intranasal esketamine.

Early studies in individuals with PTSD (and in some cases, comorbid MDD) found an improvement in symptoms following a single or repeated intravenous ketamine infusion (the standard route of administration) (Dadabayev et al., 2020; Feder et al., 2014, 2021). Subsequently, there have been larger studies (involving over 100 participants) on the ability of repeated ketamine infusion to alleviate symptoms in treatment-resistant veterans with PTSD or comorbid PTSD and MDD. One of these studies recently reported a reduction in depression, but not PTSD symptoms following treatment, albeit in comparison with an unusually strong placebo response (Abdallah et al., 2022). On a smaller scale, there has been work examining ketamine’s effects on epigenetic DNA methylation factors (NCT05294835) as a means to identify markers of therapeutic efficacy in PTSD (Yang et al., 2021). GAD symptoms (GAD-7) were found to be reduced by intravenous ketamine in a large community sample (Oliver et al., 2022). Regarding intranasal esketamine, two studies reported some benefit in treatment-refractory GAD and/or SAD (Glue et al., 2020a) and on symptoms of PTSD in individuals with co-morbid MDD (Artin et al., 2022). Ketamine is also discussed to be potentially helpful in treatment-refractory anxiety disorders such as GAD and SAD (Glue et al., 2017, 2018, 2020b; Tully et al., 2022; Whittaker et al., 2021).

Ketamine has been shown to facilitate fear extinction in rats that were subjected to stress (Paredes et al., 2022; Sala et al., 2022) or exhibiting high anxiety-like behavior (Fortress et al., 2018), but there have been positive and negative effects reported in ‘normal’ rats (Clifton et al., 2018; Girgenti et al., 2017). These behavioral effects of ketamine are associated with neural changes including increased synaptic plasticity and mTORC1 levels in the PFC, and reversal of PFC glutamatergic neuronal hyperexcitability and dendritic atrophy (Fortress et al., 2018; Girgenti et al., 2017; Paredes et al., 2022; Sala et al., 2022). In an interesting parallel from human functional imaging work, ketamine increased vmPFC-amygdala connectivity and reduced amygdala responses to threat (Krystal et al., 2021). The data raise the prospect of using ketamine as an adjunct to facilitate extinction-based exposure therapy. There are some preliminary clinical results with intravenous ketamine suggesting this combined approach may bring benefit (Pradhan et al., 2017; Shiroma et al., 2020), with further phase II trials underway (NCT04889664). A relatively large trial in which ketamine was repeatedly infused over two weeks reported a higher response rate and larger reduction in CAPS 5 scores, as compared to an active placebo, midazolam (Feder et al., 2021). Attempts to combine ketamine and psychotherapy is another example of where it will be important to draw upon the preclinical literature for ideas on how to optimize the timing of two treatment, relative to one another, for maximal therapeutic gain.

### 5.2. Other glutamatergic targets

Beyond ketamine, there are other NMDA-R antagonists being considered for their utility in anxiety and PTSD. These include two anesthetic gasses with NMDA-R antagonist properties. Nitrous oxide is being studied in comparison to midazolam in PTSD (NCT04378426), and a device (NBTX-001) delivering a subanesthetic mixture of xenon gas is the subject of trials in PTSD (NCT03635827) and PD (NCT04432155) following preliminarily reports of benefit in PD (Dobrovolsky et al., 2017). Another option is to target specific NMDA-R subtypes with a positive allosteric modulator (PAM). NYX-783 acts as a glutamate co-ligand to positively modulate NMDA-R activity, preferentially those receptors containing the 2B subunit (Lee et al., 2022). NYX 783 received a Fast Track FDA designation for the treatment of PTSD and a recently completed phase II trial indicates efficacy on some but not all outcome measures (NCT04044664). A multi-center phase IIb trial is being planned.

The therapeutic potential of the tuberculostatic drug, D-cycloserine (DCS), acting as a partial NMDA-R agonist at the glycine site has been discussed for several years, driven by the ability to facilitate fear extinction in preclinical studies (Ressler et al., 2004; Richardson et al., 2004). Coupling DCS with exposure-based therapy, mainly in PTSD, but also in specific phobia, PD and SAD, has however painted a mixed picture (Smits et al., 2020), with meta-analyses demonstrating low-to-modest but statistically significant efficacy (Bürkner et al., 2017; Mataix-Cols et al., 2017). Nonetheless, research on this topic is continuing (e.g. (Difede et al., 2022; Inslicht et al., 2021) and some authors maintain the drug’s potential remains to be fulfilled (Rosenfield et al., 2019).

Beyond NMDA-R, glutamatergic neurotransmission can be attenuated by inhibiting voltage-gated sodium channels with drugs such as riluzole (FDA approved as a treatment for amyotrophic lateral sclerosis) (Sanacora et al., 2022). The effects of riluzole on symptoms of PTSD and associated hippocampal markers are currently being investigated (NCT02019940, NCT02155829) and preliminary evidence has indicated an effect on antidepressant-resistant hyperarousal symptoms in PTSD (Spangler et al., 2020). Finally, an early open-label pilot study found therapeutic benefit in individuals with GAD (Mathew et al., 2005), whereas a multi-center, randomized phase III trial with the tripeptide prodrug conjugate of riluzole, troriluzole (BHV-4157), reported no signal in GAD (NCT03829241).

## 6. Cannabinoids: Synthetics and synaptic augmentation

Opportunities for targeting the endocannabinoid (eCB) system as a means to reduce anxiety and alleviate the symptoms associated with trauma have been extensively discussed elsewhere (Araque et al., 2017; Lutz et al., 2015; Mayo et al., 2022; Patel et al., 2017). Much of the discussion centers on two major eCBs, anandamide (AEA) and 2-arachidonylglycerol (2-AG), and their effects at the presynaptically-expressed cannabinoid type 1 receptor (CB1-R) (and to a lesser extent, CB2-R and the transient receptor potential vanilloid type 1 (TRPV1). Reflecting the widespread distribution of CB1-R in the brain, eCBs are implicated in a broad range of behavioral processes, including anxiety and responses to stressors (Gunduz-Cinar, 2021; Maldonado et al., 2020).

### 6.1. Cannabis preparations

Cannabidiol (CBD) and Δ^9^-tetrahydrocannabinol (THC) are naturally abundant in the resin of the marijuana plant. Both substances are pharmacologically active: THC acts a partial CB1-R agonist, whereas CBD has antagonist/inverse agonist effects at the CB1-R in addition to other anxiety-relevant actions, including effects at TRPV1, 5HT1A-R and peroxisome proliferator-activated receptor gamma (PPAR-γ) (Melas et al., 2021). A CBD-containing solution is FDA-approved as an antiepileptic for Lennox-Gastaut syndrome and Dravet syndrome. However, though CBD is promoted by some commercial interests as a panacea to all manner of ills, it has extensive first-pass hepatic metabolism and evidence from the rigorous evaluation of their efficacy in anxiety and PTSD has so far proven equivocal. Two small double-blind trials reported CBD-containing cannabis oil produced a therapeutic signal in SAD (Bergamaschi et al., 2011; Masataka, 2019), and a phase I trial for SAD is in the recruiting stage for RLS103, an inhaled CBD formulation that may improve delivery to the circulation and CNS (Devinsky et al., 2021). Another small double-blind, randomized phase II trial will evaluate the effects of oral CBD combined with multiple prolonged psychotherapy sessions in individuals with PTSD (NCT05132699).

Of further note in the context of synthetic cannabinoids is research on THC analogs that are FDA-approved as antiemetics, among other indications. Nabilone produced a beneficial effect on nightmares in PTSD (Jetly et al., 2015), and in a phase I for GAD, but not in a phase II follow-up (reviewed in (Turna et al., 2017)). The available evidence is more robust for dronabinol. Dronabinol has been shown using a randomized, double-blind design to facilitate fear extinction in healthy participants and produce long-term strengthening of mPFC-amygdala coupling in healthy and PTSD-diagnosed individuals (reviewed in (Mayo et al., 2022)). Clinical trials are being planned to further test dronabinol’s effects on fear learning and extinction, as well as on nightmares, in individuals with PTSD (NCT04080427, NCT04448808, NCT05226351).

### 6.2. Inhibiting eCB degradation

There are some interesting characteristics of eCB signaling that present a potentially tractable approach to pharmacologically manipulating eCBs for therapeutic gain. First, eCBs are produced and released ‘on demand’ – i.e., are recruited under conditions of requisite synaptic activity – and second, once released, a set of enzymes (fatty acid amide hydrolase (FAAH), monoacylglycerol lipase (MAGL), cyclooxygenase-2 (COX-2) are mobilized to degrade eCBs and thereby refine their synaptic actions (Katona and Freund, 2012).

Accordingly, pharmacologically inhibiting eCB-degradation allows for the augmentation of endogenous eCBs released under physiologically relevant conditions, such as exposure to stress or fear extinction, and may thereby bypass some of the unwanted effects of non-specifically activating eCB receptor signaling (reviewed in (Ozge Gunduz-Cinar et al., 2013; Mayo et al., 2022; Patel et al., 2017; Spohrs et al., 2021)). Based on preclinical and human experimental evidence that inhibition of FAAH (resulting in increased AEA levels) attenuates responses to stress and facilitates fear extinction (e.g., (Gunduz-Cinar et al., 2013a,b; Mayo et al., 2020a, 2020b)), a randomized, multi-center, double-blind phase II trial is currently evaluating the efficacy of the FAAH inhibitor, JZP150 (PF-04457845), in PTSD (NCT05178316). A 2-AG increasing MAGL inhibitor, Lu AG06466, is also in phase I for PTSD (NCT04597450), amongst other indications.

## 7. Neuropeptides: A nose for neuromodulation

The more than 100 known endogenous neuropeptides represent the largest class of signaling molecules in the nervous system (Hökfelt et al., 2018). Neuropeptides are neuronally synthesized and released messenger molecules that modulate neuronal activity via binding to G protein-coupled receptors. A large literature has shown that various neuropeptides, including corticotropin-releasing factor (CRF), cholecystokinin (CCK), arginine vasopressin (AVP), oxytocin, orexin, neuropeptide Y (NPY), neuropeptide S (NPS), galanin, and neurokinins, such as substance P, affect anxiety-related behaviors. Given neuropeptides are neuromodulators, it has been posited that peptide-acting drugs could have fewer side effects than drugs targeting major neurotransmitters such as GABA and glutamate (Gupta and Prabhavalkar, 2021; Holmes et al., 2003; Kupcova et al., 2022; Marvar et al., 2021; Sartori and Singewald, 2019). The caveat is that neuropeptides typically have a short half-life, poor blood-brain barrier penetration, broad receptor action, and high first pass and unstable metabolism. Approaches to circumvent these limitations include designing novel synthetic neuropeptide receptor agonist/antagonists or delivering neuropeptides intranasally.

The intranasal route has been considered for two neuropeptides, NPY and oxytocin. The 36-amino acid neuropeptide, NPY, is the most abundant CNS neuropeptide and signals via five receptor subtypes (Reichmann and Holzer, 2016). In preclinical rodent studies, NPY reverses the anxiogenic-like effects of CRF and inhibits excitatory neurotransmission, norepinephrinergic activity and amygdala reactivity to stressors (Chen et al., 2020; Reichmann and Holzer, 2016). These data suggest an anxiolytic profile of NPY. Military interest in NPY was kindled by research in soldiers indicating that NPY enhances stress resilience (Morgan et al., 2000). Accordingly, low CSF NPY levels were found to be associated with PTSD symptom severity (Tural and Iosifescu, 2020). Initial (phase I) clinical evidence indicates that a high dose of intranasal NPY reduces anxiety in individuals with PTSD (Sayed et al., 2018), and there is another active PTSD trial underway (NCT04071600).

The nonapeptide oxytocin is formed in the hypothalamus and secreted by the neurohypophysis. In addition to having a key role in uterine contraction and lactation, oxytocin is released in response to stressors and has anti-anxiety-like and fear extinction facilitating effects in rodents (Gunduz-Cinar et al., 2020; Jurek and Neumann, 2018). Rapid anxiolytic effects of intranasal oxytocin have been observed in non-clinical human participants, albeit in a manner dependent upon the chronicity of administration and inter-individual variables including age, gender and psychosocial functioning (Naja and Aoun, 2017; Neumann and Slattery, 2016). Variability in response magnitude across individuals has also characterized the behavioral and neural (e.g., amygdala and PFC activity) effects of intranasal oxytocin in small-scale PTSD studies to-date (reviewed in (Peled-Avron et al., 2020; Sandra Szafoni, 2022)). These findings raise questions about the broad applicability of oxytocin as a treatment for PTSD, though a more positive take is that oxytocin may be suited to more personalized medicine. Two ongoing phase II PTSD studies (NCT05207436, NCT04523922) investigating intranasal oxytocin as an adjunct to prolonged exposure therapy or brief cognitive behavioral therapy will hopefully help clarify matters.

Another nonapeptide, AVP, is also produced in the hypothalamus and secreted by the neurohypophysis. In the brain, AVP binds the V1a and V1b receptor subtypes and has been suggested to produce anxiety in PTSD and in substance-induced panic (reviewed in (Sartori and Singewald, 2019)). A randomized, double-blind proof of concept study in healthy participants found evidence of anxiolytic effects of a brain-penetrant V1a receptor antagonist, SRX246, using startle and a threat test as readouts (Lago et al., 2021). This finding is being followed-up by a phase II multi-center study evaluating the small molecule selective V1a antagonist RG7314 (balovaptan) in PTSD (NCT05401565); though it should be noted that recent trials with balovaptan in autism have proven disappointing (Hollander et al., 2022). It also seems likely that, as in the case of oxytocin, biological sex could turn out to be a major determinant of these effects (Bredewold and Veenema, 2018; Li et al., 2016).

The hypocretins consisting of the 33-amino acid orexin-A and the 28-amino acid orexin-B are produced in a small number of neurons in the lateral-dorsomedial hypothalamus but innervate large parts of the brain. Orexin signaling at its OX1-R and OX2-R receptors is strongly implicated in arousal, sleep and circadian rhythms (the orexin receptor antagonists suvorexant and daridorexant are approved insomnia treatments), but also in anxiety (Summers et al., 2020). There is preliminary evidence for a possible anxiolytic potential and arousal attenuation for OX1-R antagonism. For example, a translational study in rats and male human volunteers found that the selective OX1-R antagonist, JNJ-61393115, decreased panic symptoms induced by CO_2_ challenge (Salvadore et al., 2020). Anxiolytic effects (attenuation of response to unpredictable threat) of suvorexant in healthy volunteers have been observed (Gorka et al., 2022) and a trend for lower anxiety scores was reported with the OX-R antagonist, ACT-539313, in healthy men and women (Kaufmann et al., 2021). Although more research is planned (Jacobson et al., 2022; Yaeger et al., 2022), there are no active clinical studies with orexin antagonists in anxiety drug development although they may be conducted within the pipeline of the double orexin receptor antagonist (DORA), saltorexant.

## 8. Phytopharmaceuticals, nutraceuticals and the microbiome

There is renewed interest in using so-called alternative medicines and natural remedies to alleviate symptoms related to anxiety and trauma. Phytochemicals, substances derived from plants such as terpenes, alkaloids, flavonoids, phenolic acids, lignans, cinnamates, and saponins, have demonstrable effects on various neurotransmitters and inflammatory processes (Farzaei et al., 2016; Sarris et al., 2022). There are preclinical animal data and human reports of anxiolytic-like effects of plants including lavender, Kava, chamomile extract, saffron extract, and Galphimia glauca (reviewed in (Sartori and Singewald, 2019; Zhang et al., 2022)). Though concrete clinical evidence remains scarce, the lavender preparation, Silexan, is approved in Germany as a treatment for subsyndromal anxiety symptoms and is the subject of ongoing clinical trials in patients with mild to moderate depression (EudraCT number 2020-000688-22). Other phytochemicals, such as Kava, are also being evaluated (Soares et al., 2022), despite disappointing phase III results in GAD (Sarris et al., 2021).

One working hypothesis is that antioxidant and anti-inflammatory properties of certain plants can have therapeutic benefits (Fedoce et al., 2018; Felger, 2018; Kim et al., 2020; Nikolova et al., 2021; Won and Kim, 2020) (see discussion below on inflammation). A related area of burgeoning interest in psychiatry is the microbiome, particularly the gut microbiome and its interactions with the CNS (Dinan and Cryan, 2017; Lee and Kim, 2021; Offor et al., 2021; Simpson et al., 2021). This has fostered the study of substances ingested from food (‘nutraceuticals’) that might affect anxiety in part by altering the microbiome (Adan et al., 2019; Burokas et al., 2017; Norwitz and Naidoo, 2021; Weeks et al., 2021). The probiotic Lactobacillus rhamnosus GG is being investigated in a phase II PTSD trial for its anti-inflammatory and immunoregulatory properties. A phase I, small open-label trial investigates the effects of a novel gut microbiome therapeutic, Microbial Ecosystem Therapuetic-2 (MET-2), in GAD and MDD patients (NCT04052451).

## 9. Other systems of note

### 9.1. Hormones

Glucocorticoid (GR)-based approaches to treating PTSD have been quite extensively studied, notably as exposure therapy adjuncts to facilitate extinction (reviewed in (de Quervain et al., 2019; DePierro et al., 2019)). For example, cortisol (in the form of hydrocortisone) or the anti-inflammatory cortisol analogue, dexamethasone, have been shown to enhance fear extinction in individuals with full or subsyndromal PTSD and increase activation in extinction-mediating PFC areas (Inslicht et al., 2021; Merz et al., 2018; Michopoulos et al., 2017a; Sawamura et al., 2016). The extinction-promoting effects of hydrocortisone appear to be more pronounced in individuals with higher basal GR sensitivity (Lehrner et al., 2021). An ongoing study is investigating whether hydrocortisone in a subgroup of individuals with PTSD exhibiting HPA-axis dysregulation, improves extinction (EudraCT, 2020-000712-30). It is also worth mentioning that another steroid hormone, estradiol, is being studied at the symptom and neural (fear--related brain activity) level as an adjunct to exposure therapy in women with PTSD (NCT04192266). Taking a different tack, a double-blind, randomized phase II trial, is examining whether hydrocortisone given in the emergency room during the first hours after a traumatic experience reduces subsequent development of PTSD (NCT04924166).

### 9.2. Cholinergic system

A line of research targeting the cholinergic system is based on the observation that antagonists at the α7-nicotinic acetylcholine receptor subtype (nAChR) have anxiolytic-like properties in rodents (Mineur et al., 2016). Clinically, the α7-nAChR negative allosteric modulator, BNC210, has been shown to reduce self-reported anxiety (STAI) in individuals with GAD (Perkins et al., 2021) and decrease amygdala responses to threat (fearful faces) (Wise et al., 2020). BNC210 has been fast-tracked by the FDA for evaluation and is the subject of two multi-center, randomized phase II trials for PTSD (NCT04951076) and SAD (NCT05193409). Another phase II is examining the partial α7 nAChR agonist VQW-765 (aka AQW051 (Feuerbach et al., 2015) for ‘Performance Anxiety’ (NCT04800237).

### 9.3. Neuroinflammation

One area of increasing recent interest concerns neuroinflammation. This interest is based on data showing that inflammation influences function in emotion-regulating parts of the brain (Ravi et al., 2021) and the finding that many of the compounds discussed above (including antidepressants, cannabinoids, phytochemicals, nutraceuticals, and microbiome-targets) have anti-inflammatory components that are posited to contribute to their therapeutic actions (Hou et al., 2017; Santos et al., 2018; VanderZwaag et al., 2022). There is also evidence of changes in inflammatory signaling/markers (e.g., cytokine dysbalance, elevated inflammatory cytokines, acute-phase proteins) and the immune system and individuals with anxiety disorders and PTSD, and high trait anxiety (Costello et al., 2019; Felger, 2018; Katrinli et al., 2022; Kim and Jeon, 2018; Michopoulos et al., 2017b; Peruzzolo et al., 2022; Renna et al., 2018; Rooney et al., 2020). In a topical example, levels of certain immunological markers correlate with self-rated anxiety and depression in COVID-19 patients for several months after infection (Mazza et al., 2020, 2021). It may turn out that targeting individuals with evidence of immune over-activation will be a key to achieving therapeutic effects when using anti-inflammatory compounds, as was found to be the case in MDD with the TNF-α blocker, infliximab (Karson et al., 2013). However, the identification of biomarkers of (pro-)inflammatory signaling continues to be challenging, including in those individuals with defined immunological perturbations (e.g., (Kennedy and Niedzwiedz, 2022; Maples-Keller et al., 2022b; Won and Kim, 2020)). Nevertheless, studies are underway to evaluate the efficacy of anti-inflammatory treatments in anxiety disorders and PTSD (Elnazer et al., 2021; Maier et al., 2020; Makunts et al., 2018).

In addition to these and the other neurotransmitter and neuro-modulatory systems discussed above, numerous other mechanisms and targets have been proposed for their potential as anxiolytics and PTSD medications based on preclinical data but have not yet progressed to substantive clinical investigation. These include the targeting of the metabotropic glutamate receptor (mGluR) (Ferraguti, 2018), melatonin (Millan, 2022), cellular/mitochondrial metabolism and oxidative stress (Bersani et al., 2020; Fedoce et al., 2018; Filiou and Sandi, 2019; Zalachoras et al., 2020), and epigenetic mechanisms (Bonomi et al., 2022; Peedicayil, 2020; Whittle and Singewald, 2014), amongst others (Maltsev et al., 2021; Pérez de la Mora et al., 2022; Raut et al., 2022b).

## 10. Convergent themes and future directions

In covering a broad swathe of preclinical and clinical work, encompassing a diverse range of neurochemical systems, several convergent themes become apparent, of which we will highlight (below) two that are most closely relate to the neuroscience of anxiety and trauma. It should be noted, however, that future progress in this field will also require solutions to a number of clinical-level issues. These include the perennial difficulty in psychiatric medicine of designing drugs that can pass the blood-brain barrier and access the brain to affect changes in behavior. We also think there should be ways to improve upon the design of clinical trials (Markowitz and Milrod, 2022) and accommodate the high placebo response rates that complicate the evaluation of novel treatments (Hodgins et al., 2018; Stein et al., 2021). A related point is that while there has been a lot of enthusiasm for the idea of ‘precision medicine’ (and some remarkable breakthroughs in other clinical areas, such as cancer), the promise of developing and delivering commercially viable individualized treatments for psychiatric disorders such as anxiety and PTSD has yet to be fulfilled. Its clinical application would first require the identification of predictive markers, indexing the involvement of specific pathomechanisms. Currently, this is not in sight in anxiety disorders, although it is well conceivable that a combination of e.g. genetic, metabolic and brain imaging techniques may lead to such approaches. For a fuller discussion of these issues and other emerging trends, see (Brehl et al., 2020; Sartori and Singewald, 2019).

### 10.1. Working with the brain’s capacity for plasticity to bring about therapeutic gain

The first of these concepts is the idea that co-opting neural processes that drive plasticity to bring about changes in the brain substrates that underlie the symptoms of anxiety disorder and PTSD. In turn, the hope is that exploiting mechanisms underlying neuroplasticity could result in therapeutic benefits. Indeed, at some level all of the neurochemical systems we have discussed are implicated to some degree in neuroplasticity. The hope is that drugs targeting these systems can produce plasticity-related therapeutic benefits that are robust, lasting, and ideally rapid in onset. Promisingly, we have seen how drugs such as ketamine are shown to be fast-acting in MDD and are being evaluated not only for their rapidity, but also their ability to produce enduring therapeutic effects, in anxiety disorders and PTSD.

A complementary approach to working with the inherent malleability of the brain is to combine medication with behavioral therapies either to improve patient compliance with the therapy (e.g., by reducing fear and anxiety in the therapy setting) (Krediet et al., 2020), or to strengthen experience-dependent memories formed during therapy (e.g., fear extinction) (Bukalo et al., 2014; Heinig et al., 2017; Holmes and Quirk, 2010; Marin et al., 2020; Ressler, 2020; Singewald et al., 2015). Facilitating fear extinction may be an especially powerful approach given extinction deficits are an intermediate phenotype in PTSD and some anxiety disorders (Duits et al., 2015; Ressler et al., 2022; Suarez-Jimenez et al., 2020; Wen et al., 2022) that may account for high relapse rates (Popiel et al., 2015).

### 10.2. Leveraging the remarkable advances in circuit neuroscience to improve treatments

The second theme that recurs in the current literature is the move towards designing and clinically assessing new medications for which there is an understanding not just of pharmacological, but also – to the extent it is known - neurobiological mechanism. An emphasis on mechanism goes hand in hand with a focus on pathophysiological process (e.g., fear extinction) and less on symptom (e.g., ‘anxiety’). Premised on the notion that anxiety disorders and PTSD reflect dysfunction in how these circuits and systems respond to internal and environmental stressors – resulting in the abnormalities in cognition and emotional regulation that present with these disorders – rectifying these dysfunctions should bring therapeutic benefit (Fenster et al., 2018; Ressler, 2020; Ressler et al., 2022).

A focus on mechanism can also benefit from leveraging the impressive advances being made in neuroscience (Flores et al., 2018; Hariri and Holmes, 2015; Ressler, 2020), including the development of treatments that work at the circuit-specific level (Quagliato et al., 2022; Ressler, 2020). In addition to making strides towards to defining neural circuit substrates of anxiety and stress-related behavior, the field is making rapid progress in elucidating the genetic/epigenetic, signaling and modulatory mechanisms operating in these circuits that could represent treatment-relevant druggable targets. The value of such an approach is beginning to find support in the evidence from current treatments. For instance, positive clinical effects of cognitive behavioral therapy and SSRIs are associated with demonstrable changes in brain circuits linked to cognition and emotion (reviewed in (Baumel et al., 2022; Murphy et al., 2021; Sartori and Singewald, 2019).

### 10.3. Final remarks

The enormous and growing burden of psychiatric conditions related to anxiety and trauma is a crisis for personal lives and public health systems around the world. This is a crisis that cannot be adequately mitigated by existing therapeutic options, including pharmacotherapies. Fortunately, as have endeavored to relay in this review, there is newfound momentum in the efforts underway to expand and improve upon the mediations available to patients and clinicians to ease the suffering associated with anxiety disorders and PTSD. We discussed the rationale behind pharmacologically targeting various neurochemical systems, including the monoamine, GABA, glutamate, cannabinoid, and neuropeptide systems, and summarized ongoing clinical trials assessing the therapeutic efficacy of these drugs. Finally, we underscored the importance of designing treatments that take advantage of our growing understanding of the neural circuits underlying anxiety and the effects of trauma and exploit the brain’s inherent capacity for plasticity. Although this is an area that is rapidly evolving with many outstanding questions still to be resolved, we are optimistic that the coming years can see much-needed progress in the treatment of anxiety disorders and PTSD.

## Figures and Tables

**Table 1:** Overview of compounds in clinical development for the treatment of anxiety and PTSD.

targets	GAD	PD	SAD	anxiety	PTSD
Monoamine systems (5HT, NA, DA)	psilocybin (II) MM-120 (LSD, II) AVN-101 (III) ACH 000029 (I)		MDMA (II)	psilocybin (I-II) MDMA (II)	psilocybin (II) COMP360 (psilocybin, II) BETR 001 (I) **MDMA (III)** methylphenidate (II) l-DOPA (II) TNX-102 SL (II-III) brexpiprazole (III) doxazosin (II) quetiapine (I) pimavanserin (IV) clonidine (III) dexmetomidine (I) propranolol (II)
GABA/neurosteroid system	darigabat (I)	**aloradine (III)**			PRAX-114 (II) brexanolone (II) pregnenolone (II) allopregnanolone (II)
Glutamate system	ketamine (II)	NBTX-001 (II-III)	ketamine (II)	ketamine (I)	ketamine (III) NBTX-001 (II-III) NYX-783 (II-III) N2O (II)
Cannabinoid system	cannabidiol (III)	cannabidiol (III)	cannabidiol (III) RLS103 (I-II)	cannabidiol (II)	cannabidiol (III) dronabinol (II) **JZP150 (II)** elcubragistat (I)
Neuropeptide system				oxytocin (II)	neuropeptide Y (I-II) oxytocin (II) balovaptan (II) suvorexant (IV)
Cholinergic system				VQW-765 (II)	**BNC210 (II)**
Phytochemicals	Kava (II)			Lavender	
				Kava	
Nutraceuticals	MET-2 (I) l-glutamine (II) Mulberry juice	HB-1 (II)		Lactobacillus rhamnosus GG	Lactobacillus rhamnosus GG (II) ALTO-100 (II)
Ion channels					riluzole (I) BI1358894 (II)
Hormone systems					CORT108297 (II) estradiol (III) hydrocortisone (II) insulin (II)

The phase of clinical development (I-IV) of each compound is provided in brackets. Bold lettering depicts compounds that have received breakthrough or fast-track designation by the FDA (United States Food and Drug Administration agency). *Abbreviations:* 5HT: serotonin; DA: dopamine; GABA: γ-amino butyric acid; GAD: generalized anxiety disorder; L-DOPA: levodopa (l-3,4-dihydroxyphenylalanine); LSD: lyser gic acid diethylamide; NA: noradrenaline; NO: nitrous oxide; PD: panic disorder; PTSD: post-traumatic stress disorder; SAD: social anxiety disorder.

**Table 2:** Details of compounds in clinical development for the treatment of anxiety.

Drug	Other names	Company	Main mechanisms of action	Phase	Indication	status	Ongoing trials (study name, year first posted)	Comments
Monoamine systems (5HT, NA, DA)								
Serotonergic psychodelics								
psilocybin			PAG: 5HT_2A,2C_ > 5HT_1A_	1/2	A	NYR	NCT04754061 (2021)	anxiety palliative care
				2		R	ACTRN12619001225101 (2019)	life-threatening anxiety
				2	GAD	R	ACTRN12621001358831 (2021)	
MM-120	LSD	MindMed/The University Hospital of Basel	PAG: 5HT_1A,2A,2B,2C_ > α_1A,_ D_2_ ≫D_1,3,_ α_2A_	2	GAD	R	NCT05407064(2022)	dose finding
**MDMA**			I: NET > SERT > DAT; VMAT	2	SAD	R	NCT05138068 (SAMATI, 2021)	psychotherapy
			AG: TAAR1	2	A	NYR	ACTRN12619001334190 (2019)	life-threatening anxiety
AVN-101	CD-008–0045	Avineuro Pharmaceuticals	ANTG: 5HT_7_, H_1_, α2B > 5HT_2C,2A,6,_ α_2A,2C,1B_	2	GAD	C	NCT04524975(2020)	Phase 2 successful
		Chemrar	>5HT_2B,5A,_ α_1A_ > 5HT_1A,_ H_2_, α_1D_	3		NYR	NCT04598867(2020)	
ACH 000029		Otsuka	ANTG: α_1A,1B,1D,_ 5HT_2A_	1	GAD	R	NCT05363839 (2022)	
		Ache Laboratories	PAG: 5HT_1A, 1D_					
***GABA/Neurosteroid system***								
darigabat	CVL-865	Cerevel Therapeutics	α_2/3/5_GABA_A_ PAM	1	PD	C	NCT04592536 (2020)	
**aloradine**	PH94B	VistaGen	nasal chemoreceptors	3	SAD	T	NCT05030350 (2021)	**FDA fast track**
				3		C	NCT04754802 (Palisade-1, 2021)	
				3		R	NCT05011396 (Palisade-2, 2021)	
***Glutamate system***								
cannabidiol			AG: 5HT_1A_, TRPV_1,2_, PPArγ		GAD	C	NCT05108220 (2021)	
			PAG: 5HT_2A_			C	NCT05023759 (2021)	
			ANTG: 5HT_3_	3		NYR	NCT03549819(2018)	
			ANTG/NAM: CB_1_	1	a/f	NYR	NCT05283382 (2022)	
			iAG: CB_2_	2		R	NCT04482244(2020)	disease-related
			I: FAAH	2		R	NCT04286594(2020)	
				1/2		R	NCT04726475(2021)	memory consolidation
				2		O	NCT03948074 (2019)	disease-related
				3	SAD	NYR	NCT03549819(2018)	
				3	PD	NYR	NCT03549819 (2018)	
RLS103	Cannabidiol Technosphere^®^ Inhalation Powder	Receptor Life Sciences	AG: 5HT_1A_, TRPV_1,2_, PPArγ	1b/2a	SAD	R	NCT05429788 (2022)	
			PAG: 5HT_2A_ ANTG: 5HT_3_ NAM: CB_1_ IAG: CB_2_ I: FAAH					
***Neuropeptides***								
oxytocin			AG: OT ≫ V_1A_	n.a.	A	?	NCT04320706 (2020)	
***Cholinergic system***								
BNC210		Bionomics Limited	α7n NAM, GABA_A_ M	2	SAD	R	NCT05193409 (PREVAIL, 2022)	FDA fast track
VQW-765	AQW 051	Novartis Vanda Pharmaceuticals	α7n PAG	2	PerA	NYR	NCT04800237(2021)	
***Phytochemicals***								
Lavender			I: VGCC	n.a.	A	R	NCT04934969 (2021), NCT05276505 (2022)	
			ANTG: 5HT_1A_, NK_1_			R	NCT05377983 (2022), NCT04800744 (2021)	
						R	NCT05251337 (2022), NCT04810299 (2021)	
						C	NCT04596904 (2020), NCT04260399 (2020)	aromatherapy for diverse anxiety states: procedural anxiety dental anxiety
						C	NCT04257019 (2020), NCT05369936 (2022)	
						C	NCT05334537 (2022), NCT04079309 (2019)	
						C	NCT04848350 (2021), NCT04666363 (2020)	
						C	NCT04971668 (2021), NCT04156009 (2019)	postoperative anxiety
						NYR	NCT04912531 (2021), NCT05255874 (2022)	
						?	NCT04285385 (2020) NCT04449315 (2020) ACTRN12622001129774p (2022)	
Kava			PAM: GABA_A_	2	GAD	NYR	NCT04565145 (2020)	
			I: NET, DAT, VGSC, VGCC MAO	1		C	NCT03843502 (2019)	
			AG: CB_1_	n.a.	A	NYR	ACTRN12622001226796 (2022)	
***Nutraceuticals***								
MET-2		NuBiyota	microbial ecosystem	1	GAD	C	NCT04052451 (2019)	
Lactobacillus	LGG		probiotic	n.a.	A	C	NCT04784182 (2021)	
rhamnosus GG	ATCC strain 53103					C	NCT05343533 (2022)	
	CRMTS #11272 PTS #3766							
l-glutamine			antioxidant, protein synthesis M	2	GAD	T	NCT04274114 (2020)	
HB-1		Honeybrains Biotech	undefined	2	PD	C	NCT05071430 (2021)	
Mulberry juice				n.a.	GAD	?	NCT03935061 (2019)	
***Ion channels***								
***Hormone systems***								

Bold lettering depicts compounds that have received breakthrough or fast-track designation by the FDA (United States Food and Drug Administration agency). *Abbreviations:* ?: unknown; >:greater affinity/potency; ≫: much greater affinity/potency; 5HT: serotonin; A: anxiety; α: alpha-adrenoceptor; ACTRN: Australian New Zealand Clinical Trials Registry number; AG: agonist; αn: alpha nicotinic acetylcholine receptor; ANTG: antagonist; ATCC: American Type Culture Collection; C: completed (but in most cases no results published; CB: cannabinoid receptor; D: dopamine receptor; DAT: dopamine transporter; F: fear; FAAH: Fatty acid amide hydrolase; GABA_A_: γ-amino butyric acid A receptor; GAD: generalized anxiety disorder; GG: freeze dried; H: histamine receptor; I: inhibitor; iAG: inverse agonist; LGG: Lactobacillus freeze dried; LSD: lysergic acid diethylamide; M: modulator; MAO: monoamine oxidase; MDMA: 3,4-Methylene-dioxy-methamphetamine; n.a. not applicable; NAM: negative allosteric modulator; NCT: national clinical trial; NET: norepinephrine transporter; NK: neurokinin receptor; NMDA: *N*-methyl-D-aspartate receptor; NYR: not yet recruiting; O: ongoing; OX: orexin receptor; PAG: partial agonist; PAM: positive allosteric modulator; PD: panic disorder; PerA: performance anxiety; PPAr: peroxisome proliferator-activated receptor; R: recruiting; SAD: social anxiety disorder; SERT: serotonin transporter; T: terminated; TAAR: trace amine-associated receptor; TRPV: transient receptor potential cation channel subfamily V member; V: vasopressin receptor; VGCC: voltage-gated calcium channel; VGSC: voltage-gated sodium channel; VMAT: vesicular monoamine transporter; Y: neuropeptide Y receptor.

**Table 3:** Details of compounds in clinical development for the treatment of PTSD.

Drug	Other names	Company	Main mechanisms of action	Phase	Type of trauma (sex)	status	Ongoing trials (study name, year first posted)	Comments
Monoamine systems (5HT, NA, DA)								
Serotonergic psychodelics								
psilocybin			PAG: 5HT_2A,2C_ > 5HT_1A_	2	adult trauma	NYR	NCT05243329 (2022)	
				1	adult trauma	NYR	NCT05042466 (2021)	
				1/2	COVID-19 HC	R	NCT05163496 (2021)	psychotherapy
COMP360	psilocybin formulation		PAG: 5HT_2A,2C_ > 5HT_1A_			R	NCT05312151 (2022)	
BETR 001	2-Bromo-LSD	BetterLife Pharma	5HT_2A_ AG				an IND application submitted in 2022	
	TD-0148		5HT_6,7_ ANTG					
	BOL-148							
**MDMA**			I: NET > SERT > DAT; VMAT AG: TAAR1	2	combat	R	NCT04264026 (VALLMDMA_001, 2021)	**FDA breakthrough**
				2	childbirth (f)	NYR	NCT05219175 (2022)	
				2		E	NCT05067244 (2021)	psychotherapy
				3		E	NCT04714359 (MAPPUSX, 2021)	psychotherapy
				2	veterans	NYR	NCT05173831 (MPG1, 2021)	psychotherapy
						EA	NCT04438512 (EAMP1, 2020)	psychotherapy
				2		R	NCT04030169 (2019)	psychotherapy
				3		C	NCT04077437 (MAPP2, 2019)	Psychotherapy; positive results
				2	veterans	R	NCT04784143 (2021)	psychotherapy
						E	NCT05066282 (2021)	psychotherapy
				1/2		R	ACTRN12621001078842 (2021)	psychotherapy
methylphenidate			I: DAT > NET	2		R	NCT05133804 (2021)	
					veterans	R	NCT04885257 (2021)	
l-DOPA			dopamine precursor	2	physical/sexual assault (f)	R	NCT04558112 (2020)	**ebt**
TNX-102 SL	Cyclobenzaprine sublingual	Tonix Pharmaceuticals	α_1A_, H_1_ ANTG > NET-I > 5HT_2A_ ANTG	2/3	adult trauma	R	NCT05372887 (2022)	
brexpiprazole		Otsuka	PAG: 5HT_1A_ D_2,3_, α_1A,1D_ > 5HT_2C_	3		R	NCT04124614 (2019)	drug augmentation
			ANTG: 5HT_2A,2B,7_ α_1A,1, 1D,2C_ > 5HT_6_ > α_2A,2B_, β, H_1_			R	NCT04174170 (2019)	drug augmentation
ACH 000029		Otsuka Ache Laboratories	ANTG: α_lA,1B,1D_, 5HT_2A_ PAG: 5HT_1A, 1D_	1	healthy	R	NCT05363839 (2022)	
doxazosin			α_1_ ANTG	2		R	NCT05360953 (2022)	nightmares
						O	EudraCT 2021-000319-21 (ClonDo-PTSD, 2021)	nightmares
quetiapine			ANTG: α_1_, m_1_, H_1_ > 5HT_2A_, D_2_ > D_1_ PAG: 5HT_1A_	1	veterans	NYR	NCT04280965 (2022)	prolonged exposure
pimavanserin		ACADIA Pharmaceuticals	ANTG/iAG: 5HT_2A_ > 5HT_2C_	4	veterans	C	NCT04188392 (PIP, 2019)	sleep disturbances
					veterans	NYR	NCT04809116 (2021)	insomnia
					veterans	NYR	NCT05441280 (2022)	insomnia
dexmedetomidine	BXCL501	BioXcel Therapeutics	α_2_ AG	1	combat/non-combat healthy(m)	R	NCT04827056(2021)	
						C	NCT04508166 (TRAUMA-PRO, 2020)	boosting deep sleep after trauma
clonidine			α_2_ AG	2		R	NCT05360953 (2022)	nightmares
				3	veterans	NYR	NCT04877093 (2021)	nightmares
				2		O	EudraCT 2021-000319-21 (ClonDo-PTSD, 2021)	nightmares
propranolol			β_1,2_ ANTG	2	veterans	NYR	NCT04982211 (2021)	reconsolidation block
					**lte**	R	NCT03152175 (2017)	reconsolidation block
***GABA/Neurosteroid system***								
PRAX-114		Praxis Precision Medicine	GABA_A_ PAM	2		T	NCT05260541 (2022)	failed in MDD
brexanolone			GABA_A_ PAM	1		NYR	NCT05223829 (2022)	
				4	non-veterans (f)	NYR	NCT05254405 (2022)	
				2		R	NCT04468360 (2020)	extinction facilitation
pregnenolone			steroid receptor M CB_1_ NAM, MAP_2_ ligand, PXR AG	2	veterans	R	NCT03799562 (2019)	
allopregnanolone			GABA_A_ PAM	2		R	NCT04468360 (2020)	extinction facilitation
***Glutamate system***								
ketamine			NMDA ANTG	2		R	NCT04889664 (2021)	written EBT
				2		R	NCT04771767 (2021)	online CBT
				2	veterans	R	NCT04560660 (2020)	prolonged exposure
				1	veterans	R	NCT04032301 (2019)	
				3	veterans	C	NCT04322968 (2020)	
						E	NCT04209296 (2019)	
				2		R	NCT05294835 (2022)	
NBTX-001	Xenon	Nobilis Therapeutics	NMDA ANTG	2B/3		NYR	NCT03635827 (2018)	
**NYX-783**		Aptinyx	NMDA M	2/3		C	NCT04044664 (2019)	**FDA fast track**
						R	NCT05181995 (2022)	
N2O			I: NMDA, AMPA, kainate ANTG: VGCC, n, TREK	2	veterans	T	NCT04378426 (2020)	
***Cannabinoid system***								
cannabidiol			AG: 5HT_1A_, TRPV_1,2_, PPArγ	2		NYR	NCT05269459 (2022)	
			PAG: 5HT_2A_	1/2		R	NCT05132699 (2022)	massed prolonged EBT
			ANTG: 5HT_3_	2/3	HC workers	C	NCT04504877 (BONSAI, 2020)	
			ANTG/NAM: CB_1_	2		R	NCT04197102 (2019)	
			iAG: CB_2_	2	veterans	R	NCT03518801 (2018)	
			I: FAAH	2		R	NCT04550377 (2020)	prolonged EBT
						O	EudraCT2020-003739-62 (2020)	fear extinction
dronabinol	BX-1, THC	Bionorica	CB_1,2_ PAG	2		R	NCT04448808 (2020)	nightmares
						R	NCT05226351 (DronaMemo-2, 2022)	fear conditioning
				1		R	NCT04080427 (2019)	fear extinction
**JZP150**	PF-04457845	Pfizer Jazz Pharmaceuticals Spring-Works Therapeutics	FAAH-I	2	no combat	R	NCT05178316 (2022)	**FDA fast track**
elcubragistat	Lu AG06466/ABX 1431	Lundbeck Abide Therapeutics	MAGL-I	1		R	NCT04597450 (2020)	
***Neuropeptides***								
neuropeptide Y oxytocin			Y_1,2,5_ AG AG: OT » V_1A_	1/2		?	NCT04071600 (2019)	intranasal
				2	veterans	R	NCT04228289 (2020)	intranasal
					veterans	NYR	NCT05207436 (2022)	brief CBT
					veterans	R	NCT04523922 (2022)	intranasal; prolonged exposure
balovaptan	RG7314	Roche	V_1A_ ANTG	2	adult trauma	R	NCT05401565 (2022)	
suvorexant		Merck & Co	OX_1,2_ ANTG	4	veterans		NCT03642028 (2018)	sleep disturbance
***Cholinergic system***								
**BNC210**		Bionomics Limited	α7n NAM, GABA_A_ M	2	adult trauma	R	NCT04951076 (ATTUNE, 2021)	**FDA fast track**
***Phytochemicals***								
***Nutraceuticals***								
Lactobacillus rhamnosus GG	LGG ATCC strain 53103 CRMTS #11272 PTS #3766		probiotic	2	veterans	R	NCT04150380 (2019)	
ALTO-100		Alto Neuroscience	undefined	2		C	NCT05117632 (2021)	
***Ion channels***								
riluzole		Biohaven	VGSC-I	1		C	NCT04630444 (2020)	
			NMDA ANTG					
			GluT activator					
BI1358894		BI; Hydra Biosciences	TRPV_4/5_-I	2		R	NCT05103657 (2021)	in 2020 phase I trial for drug-induced panic symptoms completed
				2		O	EudraCT2021-003154-23 (2021)	
***Hormone systems***								
CORT108297		Corcept	GR ANTG	2	veterans	R	NCT04452500 (2020)	
		Therapeutics						
estradiol			AG: ER, mER	3	(f)	R	NCT04192266 (2019)	prolonged exposure
				1	(f)	R	NCT03973229 (2019)	
hydrocortison	RVG 50730			2		R	NCT04924166 (2021)	
						O	EudraCT 2020-000712-30	safety learning intranasal
insulin			InsR AG	2		R	NCT04044534 (2019)	

The type of trauma studies is provided if specified. Clinical trials are performed in men and women unless otherwise stated in brackets in type of trauma. Bold lettering depicts compounds that have received breakthrough or fast-track designation by the FDA (United States Food and Drug Administration agency). *Abbreviations:* (f): female; (m): male; ?: unknown; >:greater affinity/potency; ≫: much greater affinity/potency; 5HT: serotonin; α: alpha-adrenoceptor; ACTRN: Australian New Zealand Clinical Trials Registry number; AG: agonist; αn: alpha nicotinic acetylcholine receptor; AMPA: α-amino-3-hydroxy-5-methyl-4-isoxazolepropionic acid receptor; ANTG: antagonist; β: beta-adrenoceptor; BI: Böhringer Ingelheim; C: completed (but in most cases no results published); CB: cannabinoid receptor; CBT: cognitive behavioral therapy; D: dopamine receptor; DAT: dopamine transporter; E: enrolling by invitation; EA: expanded access; EBT: exposure based therapy; ER: estrogen receptor; EuraCT: European clinical trials register; FAAH: Fatty acid amide hydrolase; GABA_A_: γ-amino butyric acid A receptor; GG: freeze-dired; GluT: glutamate transporter; GR: glucocorticoid receptor; H: histamine receptor; HC: health care; I: inhibitor; iAG: inverse agonist; InsR: insulin receptor; LGG: lactobacillus freeze-dried; LSD: lysergic acid diethylamide; LTE: persons exposed to life threatening events such as police officers, M: modulator; m: muscarinic acetylcholine receptor; MAGL: monoacylglycerol lipase; MAP: microtubule-associated protein; MDD: major depressive disorder; MDMA: 3,4-Methylene-dioxy-methamphetamine; mER: membrane estrogen receptor; n: nicotinic acetylcholine receptor; NAM: negative allosteric modulator; NCT: national clinical trial; NET: norepinephrine transporter; NMDA: *N*-methyl-D-aspartate receptor; N2O: nitrous oxide; NYR: not yet recruiting; O: ongoing; OT: oxytocin receptor; OX: orexin receptor; P/S physical or sexual assault; PAG: partial agonist; PAM: positive allosteric modulator; PPAr: peroxisome proliferator-activated receptor; PTSD: post-traumatic stress disorder; PXR: pregnane X receptor; R: recruiting; S: suspended; SERT: serotonin transporter; TAAR: trace amine-associated receptor; THC: delta-9-tetrahydro-cannabinol; TREK: voltage-gated potassium channel; TRPV: transient receptor potential cation channel subfamily V member; V: vasopressin receptor; VGCC: voltage-gated calcium channel; VGSC: voltage-gated sodium channel; VMAT: vesicular monoamine transporter; Y: neuropeptide Y receptor.

**Table 4:** Overview of compounds in clinical development investigated in drug-assisted psychotherapy for the treatment of anxiety and PTSD.

targets	GAD	PD	SAD	anxiety	PTSD
Monoamine systems (5HT, NA, DA)			MDMA (II)	psilocybin (I/II)	psilocybin (I/II) MDMA (II) l-DOPA (II) quetiapine (I)
GABA/neurosteroid system					brexanolone (II) allopregnanolone (II)
Glutamate system					ketamine (II)
Cannabinoid system			cannabidiol/RLS103 (I/II)	cannabidiol (II)	cannabidiol (II) dronabinol (I)
Neuropeptide system					oxytocin (II)
Cholinergic system					
phytochemicals					
Ion channels					
Hormone system					estradiol (III) hydrocortison (II)

The phase of clinical development (I-III) of each compound is provided in brackets. *Abbreviations:* 5HT: serotonin; DA: dopamine; FAAH: fatty acid amide hydrolase; GABA: γ-amino butyric acid; GAD: generalized anxiety disorder; LSD: lysergic acid diethylamide; NA: noradrenaline; PD: panic disorder; PTSD: post-traumatic stress disorder; SAD: social anxiety disorder.

## Data Availability

No data was used for the research described in the article.

## Abbreviations:

RDoc: Research Domain Criteria
